# Altered Liver Biochemistry and Mortality in Patients Hospitalized With COVID-19

**DOI:** 10.7759/cureus.54218

**Published:** 2024-02-14

**Authors:** Felipe A Muñoz Rossi, Diana Marcela Gallo Orjuela, Ana Maria Guaiquil, Camilo Gonzalez, Juanita Salazar Agudelo, Néstor Israel Quinapanta Castro, Angie Osorio, Diana Villegas Valle, Angel Moncayo Castillo, Jose Cabarcas Rua

**Affiliations:** 1 Internal Medicine, National University of Colombia, Bogota, COL; 2 Maxillofacial Surgery, Pontifical Javeriana University, Bogota, COL; 3 Medical Affairs, Nueva Granada Military University, Bogota, COL; 4 Research in Health Sciences, Centre Ophtalmologique Place de Paris, Luxembourg, LUX; 5 Emergency, Pablo Tobon Uribe Hospital, Medellin, COL; 6 Emergency, CES Clinic, Medellin, COL; 7 Research and Biostatistics, Autonomous Regional University of the Andes, Ambato, ECU; 8 General Medicine, Sinú University Elías Bechara Zainúm, Cartagena de Indias, COL; 9 Anatomic Pathology, Hospital General Babahoyo Ecuadorian Institute of Social Security, Babahoyo, ECU; 10 General Surgery, Martin Icaza General Hospital, Babahoyo, ECU; 11 Internal Medicine, University of Buenos Aires, Buenos Aires, ARG

**Keywords:** prognosis, hospitalization, death, sars-cov-2, aminotransferases

## Abstract

COVID-19 is an illness caused by the SARS-CoV-2 virus, a type of coronavirus initially identified in China in late 2019, emerging as the leading cause of death attributed to a single infectious agent worldwide. The COVID-19 pandemic poses a substantial challenge to global public health in the first quarter of this century. The rapid evolution of the pandemic and its intricate response have hindered the formulation of definitive conclusions, and it may take years to comprehend its long-term effects. Assessing the extent of organ damage beyond the lungs could guide physicians in the disease's severity or progression. Based on these characteristics, an earlier and more targeted approach can be initiated at the appropriate moment. The association between hepatic profile and mortality in COVID-19 patients is a subject of scientific interest, as SARS-CoV-2 infection can lead to hepatitis. In severe cases, it may induce sepsis-related liver injury, potentially culminating in hepatic failure.

Methodology: The study's objective is to determine the prevalence of mortality in adult patients with elevated hepatic profile hospitalized due to SARS-CoV-2 infection. This cross-sectional, monocentric study was conducted at a healthcare institution in Bogotá, Colombia.

Results: This study includes 91 patients with confirmed diagnoses of COVID-19, revealing a prevalence of hepatic profile alterations in 61.5% (n=56) of hospitalized patients. The mortality rate observed is 17.6% (n= 16), with an odds ratio (OR) of 12.4 (95% CI = 1.56-99.0) in patients with hepatic profile alterations.

Conclusions: This research underscores the importance of early detection of hepatic profile alterations in hospitalized patients with COVID-19. Not only are these alterations prevalent, but they are also potentially associated with an increased risk of mortality. These findings emphasize the necessity for further research to enhance strategies and prognostication for patients with COVID-19 in the future.

## Introduction

The COVID-19, caused by SARS-CoV-2, has emerged as a leading cause of mortality associated with common infectious agents worldwide [1]. In this first quarter century, the COVID-19 pandemic has become a formidable public health challenge, highlighting the rapid evolution of the virus and the complexity of responses encountered thus far. However, due to the swift spread of the virus and the ongoing evolution of the situation, a comprehensive understanding of its long-term effects remains to be determined. It will necessitate thorough investigations and analyses in the coming years.

A highly contagious novel coronavirus that emerged in late 2019 swiftly disseminated globally, triggering a pandemic. The virus is transmitted from person to person, both by symptomatic and asymptomatic individuals, through close contact (approximately 2 meters), respiratory droplets, aerosols, and even contact with fomites. However, the latter is not considered a primary route [2].

Most individuals infected with SARS-CoV-2 present mild symptoms, while a minority of patients require hospitalization and admission to the critical care unit, potentially leading to severe/critical illness that triggers acute respiratory failure, septic shock, and multiorgan failure [3].

The impact of SARS-CoV-2 infection on the liver has been studied, revealing various manifestations. Some individuals experience abnormalities in liver function tests, such as elevated levels of hepatic enzymes ALT and AST, indicating inadequate liver function.

The association between the hepatic profile and mortality in COVID-19 patients is a subject of scientific interest. SARS-CoV-2 infection can lead to hepatitis, and in severe cases, it may induce sepsis-related liver injury, potentially culminating in hepatic failure [3,4].

According to clinical data reports, abnormal liver function tests were relatively frequent in individuals with COVID-19 [4]. AST and ALT levels were elevated in individuals with severe disease, with a prevalence of 40% for aspartate aminotransferase (AST) and 28% for alanine aminotransferase (ALT) [5]. The cumulative incidence of liver injury in patients infected with SARS-CoV-2 can vary widely, ranging from 15% to 65%, respectively [6].

Recent studies have demonstrated that patients with preexisting liver disease are at a higher risk of developing a severe course of COVID-19 [7,8]. However, a recent meta-analysis revealed that, despite this elevated risk, the mortality rate in patients with preexisting liver disease was surprisingly low, ranging from 0% to 2%. This was significantly lower than the rates associated with other commonly studied comorbidities such as hypertension, diabetes mellitus, cardiovascular diseases, and respiratory diseases [9].

Other studies, primarily conducted in Western regions, have not identified a significant association between hepatic biochemical levels and mortality. However, some of these studies have found that elevated levels, significantly when exceeding five times the upper normal limit, are linked to an increased risk of death [8]. Clinicians could be advised on the severity or extent of the condition by estimating the degree of organ damage. Based on these criteria, an early intervention could be initiated at the appropriate time [10].

The present study aims to determine the prevalence of hepatic profile alterations in COVID-19 hospitalized patients and their frequency in those who died. According to the American College of Gastroenterology, abnormalities in liver enzymes were observed in 30% of individuals with confirmed COVID-19 infection. However, other studies report a prevalence of around 50% at admission [11,12].

This was subsequently confirmed in pathological studies of deceased patients due to SARS-CoV-2 infection, with evidence of microvesicular steatosis, focal necrosis with lymphocytic infiltration, and thrombosis in the portal area [13]. Given the alterations mentioned above, the relationship between hepatic chemistry and hospital deaths from COVID-19 remains a topic of controversy, enabling the early identification of patients at higher risk. This could have significant implications for primary care and the implementation of preventive strategies.

## Materials and methods

This retrospective cross-sectional study was conducted at a private healthcare provider in Bogotá, Colombia, focusing on 200 medical records of patients aged 18 and above hospitalized with a confirmed diagnosis of COVID-19 between February 2020 and April 2022. To achieve its objectives, the study aimed to collect sociodemographic, clinical, and specific biomarker data. Inclusion criteria encompassed patients aged 18 and above with a confirmed diagnosis of COVID-19 and hypoxemia defined by blood gas analysis parameters.

Exclusion criteria comprised pregnant patients, HIV-infected individuals, those with active malignancy, patients with acute organ injuries, transplant recipients, those undergoing emergency surgery, individuals with preexisting liver disease, patients with stage III or higher chronic kidney disease, and those with severe preexisting functional and instrumental dependence.

A sample size of 91 medical records was calculated, with a confidence level of 99% and a margin of error of 10%. A database was created to record sociodemographic and laboratory parameters, including age, gender, height, BMI, length of hospital stays, mortality, and biochemical characteristics.

Categorical variables were presented using contingency tables with frequencies and percentages. In contrast, continuous variables were displayed using measures of central tendency, such as median or mean, depending on their normal distribution as determined by the Kolmogorov-Smirnov test.

The means of continuous quantitative variables were compared using t-student tests when the data showed normal distribution; otherwise, the non-parametric Mann-Whitney U test was applied. The chi-square test was utilized for the comparison of qualitative variables. All analyses were conducted using the statistical software packages SPSS version 29.0 (IBM Corp., Armonk, NY) for Windows, R studio, and Jamovi, with statistical significance set at a level of P < 0.05.

Ethical considerations

Considering the retrospective nature of the chart review, this research is deemed low risk. No interventions that could modify patients' behaviors and treatments will be conducted. Therefore, obtaining informed consent is not necessary. Confidentiality of health information will be ensured, with no mention of patient names or involved medical personnel. For the execution of this study, approval was sought from the relevant ethics committee of the healthcare institution, adhering to the guidelines of good clinical practice.

## Results

The present investigation included 91 individuals with a confirmed diagnosis of SARS-CoV-2 infection, determined through a positive polymerase chain reaction (PCR) test. Of the total individuals, 64.8% were male, and 35.2% were female. The mean age was 51 years. Among the patients, 51.6% (n=47) were older than 50, 30% (n=27) were in the 36-50 age group, and only 18.7% (n=17) were younger than 35.

None of the individuals had a history of prior liver disease. The mean duration of hospital stay was 6.83 days (95% CI: 5.26-8.40), and the mortality was 17.6% (n=16), as described in Table 1.

**Table 1:** Demographic characteristics

Variables		n=91
Age (mean)		51 (CI 95% 48.0-54.0)
Gender		
M (%)		59 (64.8%)
F (%)		32 (35.2)
Height (mean)		166 (SD 9.41) (CI 95% 164.1-168.1)
Weight (mean)		81.7 (SD 17.81) (CI 95% 77.9-85.5)
BMI (mean)		28.52
Hospital stays (mean)		6.83 (CI 95% 5.26-8.40)
Comorbidities		
Arterial hypertension		
No (%)		59 (65.6%)
Yes (%)		31 (34.4%)
Dyslipidemia		
No (%)		72 (80.0%)
Yes (%)		18 (20.0%)
Diabetes mellitus		
No (%)		70 (77.8%)
Yes (%)		20 (22.2%)
Mortality		
No (%)		75 (82.4%)
Yes (%)		16 (17.6%)

Characteristics of patients with an altered liver profile in patients hospitalized for COVID-19

**Figure 1:**
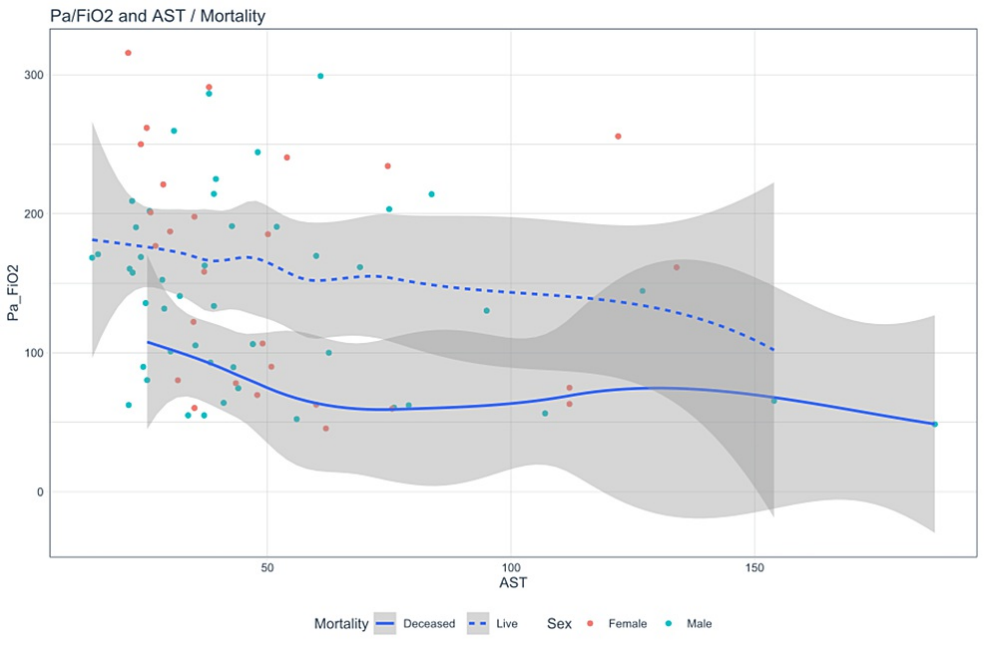
Scatterplot smoothing: correlation between oxygenation disorder and AST. AST: aspartate aminotransferase

During hospitalization, the prevalence of patients with altered hepatic profiles was 61.5% (n=56) (95% CI = 51.4%-71.1%). Moreover, a weak correlation was identified between the elevation of AST and the increase in oxygenation disorder (p=0.034) (Table 2), as detailed in Figure 1.

**Table 2:** Correlation matrix

		Oxygenation disorder
AST	R Pearson	-0.246
	P-value	0.034

Among patients with altered hepatic profiles, 41.8% (n=23/55) experienced severe oxygenation disorder, while only 23.6% (n=13/55) showed mild disorder. In other words, up to 82.1% (n=23/28) of patients with severe oxygenation disorder exhibited hepatic profile alterations (Table 3), with a statistically significant relationship (p=0.021) (Table 4).

**Table 3:** Contingency table

			Oxygenation disorder		
			Mild	Moderate	Severe
Hepatic Profile	Normal	Observed (n)	15	11	5
		% Hepatic Profile	48.4%	35.5%	16.1%
		% Oxygenation Disorder	53.6%	36.7%	17.9%
	Abnormal	Observed (n)	13	19	23
		% Hepatic Profile	23.6%	34.5%	41.8%
		% Oxygenation Disorder	46.4%	63.3%	82.1%

**Table 4:** Pearson's chi-square ^a^0 boxes (0.0%) have expected a count less than 5. The minimum expected count is 10.09.

	Value	df	P-value
Pearson's Chi-square	7.754^a^	2	0.021
N of valid cases	86		

**Figure 2:**
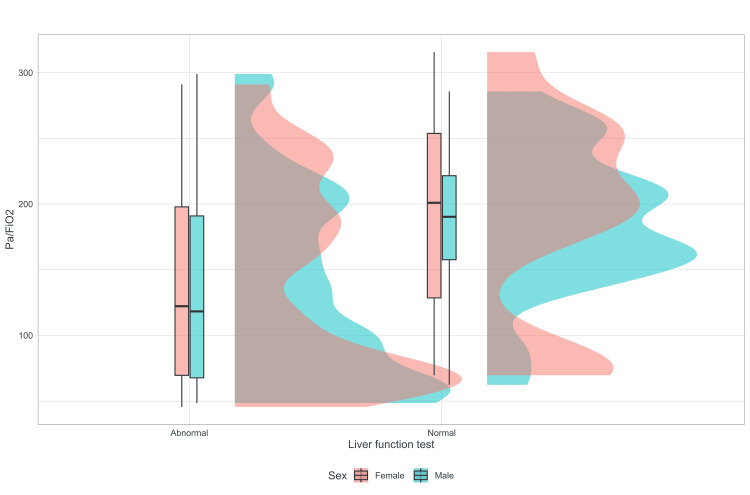
Rain cloud plot, liver function, and PA/FiO2

The patients with altered hepatic profiles concerning oxygenation disorder show a statistically significant difference, as indicated by the Mann-Whitney U statistic (p=0.002), for a variable with a non-normal distribution (Kolmogorov/p=0.598) and equal variance (p=0.158) (Figure 2).

**Figure 3:**
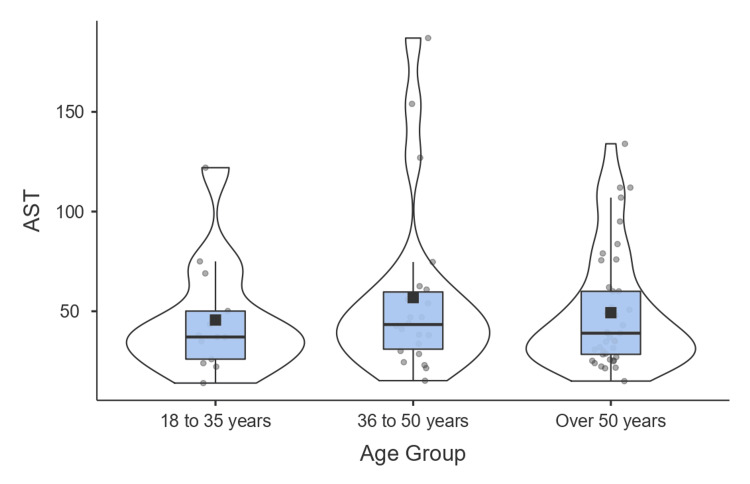
Box plot: age groups/AST AST: aspartate aminotransferase

Regarding patient groups, it was observed that those aged between 36 and 50 years had a higher elevation in AST levels, with a mean of 56.9 (95% CI 37.4-76.3) (Figure 3).

**Figure 4:**
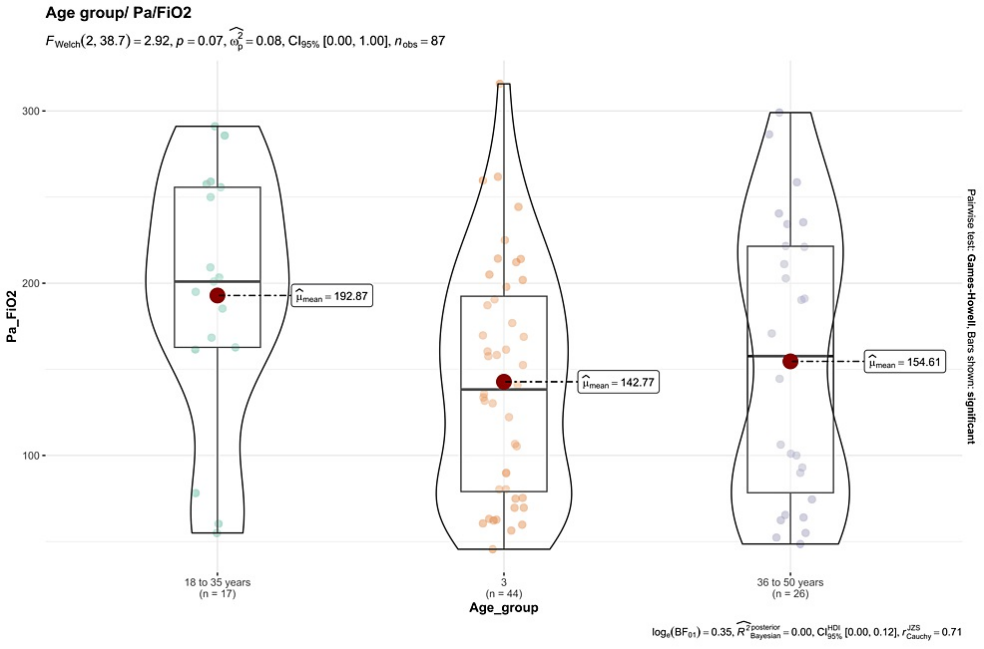
Box plot: age groups/PaFiO2

In the group aged over 50 years, patients exhibited a higher proportion of severe oxygenation disorder, with 53.6% (n=15/28) (Figure 4).

Analyze the relationship between altered hepatic profile and mortality

**Figure 5:**
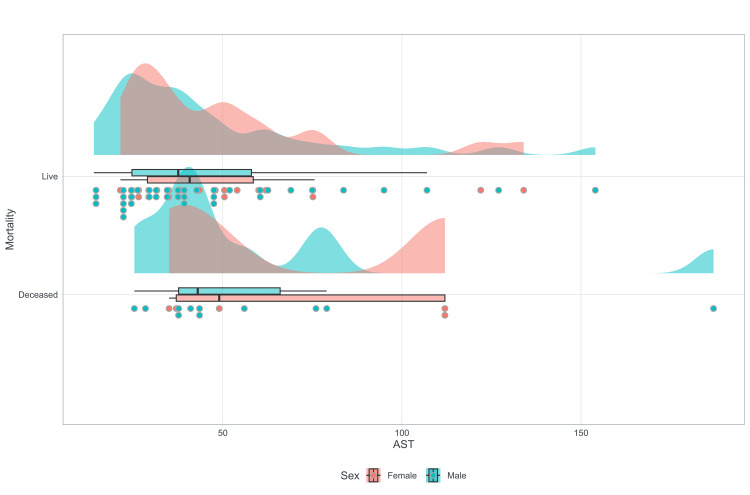
Rain-cloud plot AST/mortality AST: aspartate aminotransferase

The prevalence of mortality in our study was 17.6%, of which 93.8% had an altered hepatic profile (n=15/16) with p=0.004. Regarding the association between mortality and aspartate aminotransferase (AST) activity in this research, it is evident that mortality is higher in individuals with higher AST levels. Patients with AST levels above 100 are 2.4 times more likely to die than those below 50 (OR=2.4; 95% CI = 0.48-11.8). Additionally, it indicates that women with AST levels above 100 are two to three times more likely to die than men with AST levels above 100 (OR=3.0, 95% CI = 0.15-59.9) (Figure 5).

**Figure 6:**
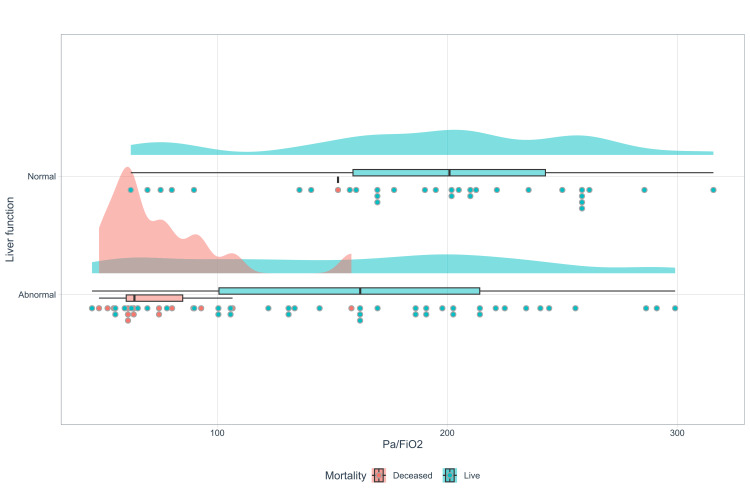
Raincloud plot liver function/PaFiO2-mortality

**Figure 7:**
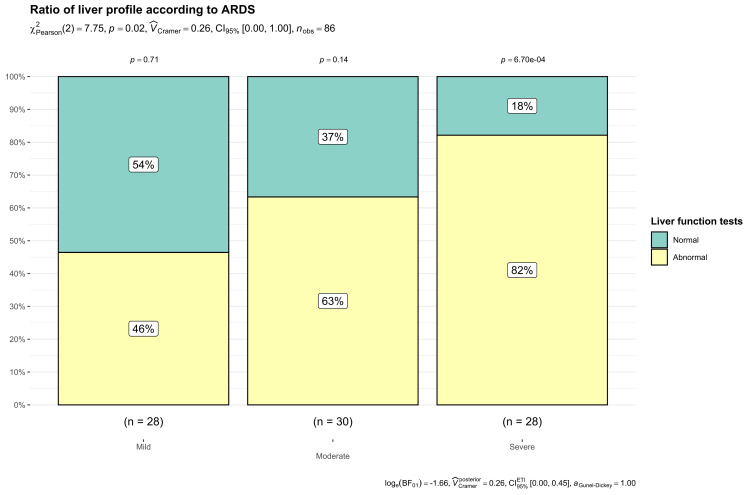
Ratio of liver profile according to ARDS ARDS - Acute respiratory distress syndrome

82.1% (n=23/28) of the patients exhibited an altered hepatic profile with severe oxygenation disorder, suggesting a potential association between decreased oxygenation and an elevation in the hepatic profile (Figures 6, 7).

The prevalence of deceased patients who had an elevated hepatic profile was 93.8% (χ²=8.51, p=0.004) with a statistically significant difference between the groups, with a prevalence ratio of 14.88 and OR=12.4 (95% CI=1.56-99.0) (Table 5).

**Table 5:** Liver function and mortality contingency table

		Hepatic Profile		
Mortality		Normal	Abnormal	Total
No	Observed (n)	34	41	75
	% Row	45.3 %	54.7 %	100.0 %
Yes	Observed (n)	1	15	16
	% Row	6.3 %	93.8 %	100.0 %
Total	Observed	35	56	91
	% Row	38.5 %	61.5 %	100.0 %

**Figure 8:**
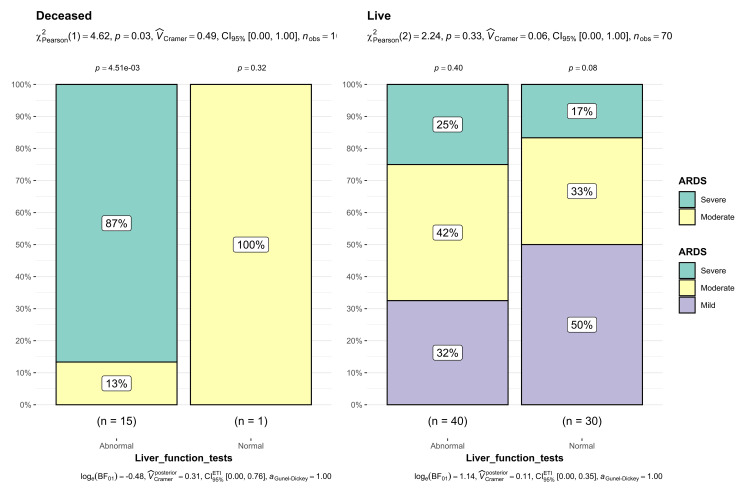
Proportion of oxygenation disorder and liver profile in terms of mortality

This study reveals a significant association between altered hepatic profile and the severity of oxygenation disorders in patients, where 87% (n=13) of the deceased patients exhibited severe oxygenation disorder compared to 25% (n=10) of the patients who survived. These findings suggest that severe oxygenation disorder and an alteration in the hepatic profile may be a statistically significant and crucial prognostic factor (p=0.03) (Figure 8).

## Discussion

This research focuses on patients with COVID-19 and provides a better understanding of the relationship between hepatic profile alterations, oxygenation disorder, and mortality. It also offers insight into the complexity of this disease and its systemic effects.

A predominance of men among COVID-19-affected patients was observed, reaching 64.8%, likely due to a higher frequency of comorbidities, which could be a confounding factor when determining gender proportions.

One key finding of this study is the prevalence of 61.5% of patients with hepatic profile alterations during hospitalization, of which 41.8% had severe oxygenation disorder, with a more significant impact in the group aged over 50, with a proportion of 53.6%. These findings are like other reported studies, such as those conducted by Yao et al. [14], who found a proportion more significant than 50%, like Fan et al. [12]. Therefore, the prevalence of liver injury varies from 9.8% [15] to 61.5%. As evidenced in this research, this may be influenced by various variables such as patients' baseline characteristics, including age, body mass index, severity of oxygenation disorder, preexisting comorbidities, and multiorgan failure.

Existing research indicates a reciprocal connection between the virus and the hepatic system. A modest but statistically significant relationship between increased AST levels and oxygenation disorder underscores the interconnection between these factors. This finding highlights the need to include hepatic profile assessment as a crucial component in the clinical evaluation of these patients.

Considering the above, 82.1% of those with severe oxygenation disorder presented hepatic alterations, emphasizing the relevance of active monitoring of hepatic function in critical patients. This significant association (p=0.021) suggests that the hepatic profile could indicate disease severity early.

In a recent study, Ding et al. found a mortality rate of 9.7% in a sample of 2,073 subjects, with around 62% presenting some hepatic profile alteration [13]. Consistently, our study highlights how AST levels increase in relation to oxygenation disorder and the risk of mortality.

This study's 17.6% mortality rate emphasizes the importance of COVID-19 infection. It is noteworthy that 93.8% of deceased individuals had hepatic function alterations, indicating a significant connection between liver disease and death. The correlation between mortality and AST levels highlights the significant predictive value of this indicator, especially in women with levels exceeding 100, who have a higher likelihood of death. These results underscore the importance of considering liver function in managing hospitalized patients, providing relevant information for decision-making and possible therapeutic interventions.

The main limitation of this study is the use of odds ratio (OR) as a measure of association. Therefore, it is relevant to consider that the OR tends to overestimate the effect of hepatic profile in relation to the outcome of interest, in this case, mortality. Since the OR can be influenced by various factors, including the prevalence of liver injury and the unique characteristics of the studied population, this overestimation may affect the interpretation of the true magnitude of the association between hepatic profile and mortality. Therefore, any conclusion based on the OR should be approached with caution due to its intrinsic tendency to exaggerate the impact of the hepatic profile on mortality.

Prior registration of the data is carried out to mitigate selection bias, mainly non-response bias, analyzing the quality of responses to the variable of interest. This will allow the data to be comparable between groups. For information bias during follow-up, the same information collection protocol is implemented for both groups, including collecting biochemical variables using the same techniques, such as ELISA and semi-automatic systems.

Additionally, a pilot test of the selected variables was conducted in 20 subjects with the collection of information, and adjustments were made based on their results to ensure complete data collection and evaluate the quality of information sources. Observer bias in participants, given the study's retrospective nature, is largely minimized.

A stratification of sociodemographic and pathological history data is carried out, coupled with a multivariate analysis using the statistical package SPSS version 29.0 to mitigate confounding bias significantly. Additionally, information for each subject will be recorded in the data collection format, and prior to digitization, the quality and plausibility of each data point will be evaluated.

## Conclusions

This research emphasizes the need to promptly and consistently identify hepatic function in individuals with COVID-19. Hepatic profile alterations are not only prevalent but also associated with the severity of the disease and possibly an increased risk of death. These findings underscore the need for specific monitoring protocols and new strategies to improve the prognosis of patients with COVID-19.
